# Apert Syndrome: Report of a rare congenital malformation

**DOI:** 10.12669/pjms.333.12878

**Published:** 2017

**Authors:** Ehsan Rathore, Altaf Hussain Rathore

**Affiliations:** 1 Dr. Ehsan Rathore, BDS, MCPS (MFS). Senior Registrar, Maxillo Facial Surgery, Faryal Dental College, Lahore, Pakistan; 2 Prof. Dr. Altaf Hussain Rathore, MBBS, D.A (London) FRCS (Edin). Chief of Surgery, Foundation Hospital Rajana, Distt: Toba Tek Singh, Punjab, Pakistan

**Keywords:** Apert syndrome, Rare congenital anomaly

## Abstract

A rare case of an adult male with malformation of the skull, face, hands and feet called acrocephalosyndactly or Apert syndrome is presented. Its probable cause, features and treatment is discussed. It is a unique case who survived upto the age of 32 years without any operative intervention and adjusted in the society though he has all the stigmas of the above syndrome. We have concluded and made a point that in the adult sufferer, facial deformity is not so important and urgent for the treatment than syndactyly, which handicaps the sufferer in performing the daily routine work.

## INTRODUCTION

Apert syndrome is a rare congenital disorder. This syndrome was first described by Eugene Apert in 1906. It is caused by a defect on the fibroblast growth factor receptor 2 gene on chromosome 10.^1^ It is an autosomal dominant disorder or more commonly due to mutation in the F.G.F.R 2 gene.^2^ It is characterize by early union of the coronal suture giving typical skull and facial deformity and sydactaly of hands and feet.^3^

It occur in 1 in 200,000 to 1 in 160,000 live births^4^,^5^ but its incidence is 1 in 65000.^6^ It is characterised by high prominent forehead flat back of occiput, flat or concave face due to premature union of coronal suture and deficient growth of midfaical bones leading to mandibular prognathism. Patient has also shallow orbit causing some degree of proptosis and hypertelorism.^3^ They may be accompanied by fusion of cervical vertabra specialy C5-6^7^ and other visceral, skeletal C.N.S anomalies.^8^

The main difference from Cruzon syndrome is that it is accompanied by syndactyly of hands and feet.^9^ Its proper treatment is operation within first year of Birth^10^ to stop early fusion of the coronal suture otherwise they get increased intracranial pressure. They have difficulty in breathing causing sleep Apnea and mouth breathing.^11^ They have usually middle ear infection resulting into some degree of deafness and can also have some degree of visual loss.^8^ They are somewhat mentally subnormal and have dentition problems. Their height is shorter than general population due to short limbs but not as short as achondroplasia.^8^ As stated earlier those who survive with or without some sort of coronal suture surgery need maxillaofacial surgeon for advancement of fronto facial and mid facial part of skull to correct the proptosis and for cosmetic reason. Syndactyly has to be operated in stages within 1-2 years age^10^ by hand or plastic surgeon in type 3, 4 for functioning of the hand specially thumb and little finger Syndactyly of feet is usually left alone.

## CASE REPORT

**Fig.1:**
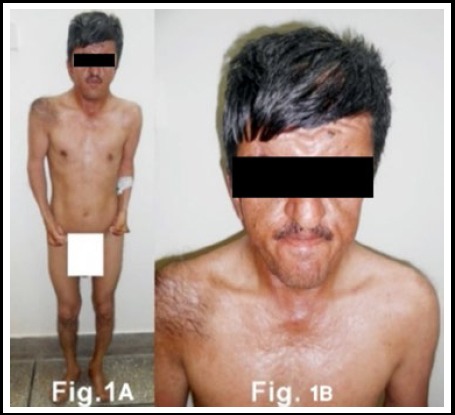
1A. Patient with Apert Syndrome. 1B. Face of the case.

**Fig.2:**
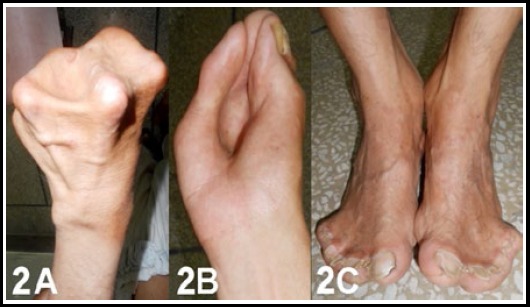
2A. Hoof shaped Hand. 2B: Syndactyly shape of a rose bud. 2C: Syndactyly of feet.

A 32 year old short statured male (Fig.1) with low intelligence building and red eyes presented to us for syndactaly of both hand and syndactyly of feet. Fig.2-A,B,C. He had low intelligence and could not tell about his pediatric and birth history as his parents died long ago. However no one else in the family had this defect. With this deformity of hands, he could not do the daily routine work. Therefore, he was more interested in operation of the hands. His face, eyes, head and teeth were classical of craniosyntosis as describe earlier.

**Fig.3:**
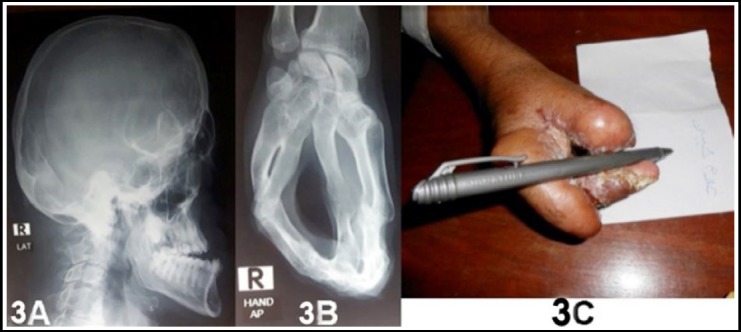
3A: X-Ray Skull. 3B: X-Ray of the hand. 3C: Right hand after the operation.

X-ray of skull (Fig.3A) was also typical of apert syndrome and syndactyly of both hands was of type III i.e fusion of all digits like a hoof or rosebud (Fig.3B) by cartilage with one conjoined nail. Foot syndactyly was of type IV.^12^

He was not bothered about his cosmetic appearance but was more concerned about hand, so facial deformity was left for the first author (E.R) to be corrected later on.

### Operation

After proper investigations he was operated under General Anesthesia. Tourniquate of arm was applied. As the blood supply of neighboring finger was complicated and construction of thumb of enough length with proper first web space was desired so we had to amputate the second digit. Right hand was operated in the first stage, construction of the thumb and middle finger was done (Fig.3C) Separation of other fingers was left to be managed later. Patient was discharged after 10 days of the surgery.

## DISCUSSION

We have reported a rare congenital malformation Apert syndrome or achrocephalosyndactyly. Its incidence is 1:65000, so it is not so rare; Apert himself reported 9 cases in 1906.^6^ They usually die in infancy unless they are operated for coronal suture in early infancy.^13^ Exact number of cases reported to date are not known. Our case is unusual that he survived upto the age of 35 years without any operation. He has got all the minor stigmas of Apert Syndrome i.e low intelligence, partial deafness, weak eye sight, short stature, facial deformity, bad dentition, mandibular prognathism, fused digits of hands and feet.

At this age, he was least worried about his facial features and adjustment in the society. His main worry was about the syndactyly of his hands due to his inability to perform the daily essential work like washing, working, eating, writing etc. For adults the surgeons should give preference to his hand surgery than facial deformity unless there are some serious respiratory problems.

He had the most difficult syndactyly i.e. Type III so the patient was badly handicapped due to hoof shaped hands. In the first stage we constructed the thumb and the first web space against the advice of Zucker^14^ who recommended the release of first and fourth web space in the first stage. In view of the severity of this deformity we amputated the index finger to make a reasonable web space along with construction of middle finger, which will take the function of the index finger. Till the time of reporting the patient is satisfied with his hand and he is washing, eating and writing himself by his right hand. We are going to plan surgery of the left hand and other fingers and facial deformity later on when he will demand it.
